# Biocorrosion caused by microbial biofilms is ubiquitous around us

**DOI:** 10.1111/1751-7915.13690

**Published:** 2020-12-15

**Authors:** Wenwen Dou, Dake Xu, Tingyue Gu

**Affiliations:** ^1^ Institute of Marine Science and Technology Shandong University Qingdao China; ^2^ Shenyang National Lab for Materials Science Northeastern University Shenyang China; ^3^ Department of Chemical and Biomolecular Engineering Institute for Corrosion and Multiphase Technology Ohio University Athens OH 45701 USA

## Abstract

Biocorrosion first surfaced in the scientific literature when Richard H. Gaines associated corrosion with bacterial activities in 1910. It is also known as microbiologically influenced corrosion (MIC). In general, it covers two scenarios. One is that microbes cause corrosion directly, which usually means microbes secrete corrosive metabolites or microbes harvest electrons from a metal for respiration to produce energy. In the second scenario, microbes are behind the initiation or acceleration of corrosion caused by a pre‐existing corrosive agent such as water and CO_2_, by compromising the passive film (often a metal oxide film on a metal). MIC is caused by microbial biofilms. It is everywhere around us. This work dissects some notable examples with perspectives.

## Graphical abstract

Biocorrosion is caused by microbial biofilms. It is ubiquitous around us. We must understand it in order to treat it.

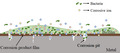

## Biocorrosion and microbial biofilms

The consensus is that 20% of all corrosion costs could be attributed to MIC, which amounts to billions of dollars in the US each year. MIC impacts various industries, such as oil and gas, water utilities, power generation and even biomedical implants. Its presence can be felt in our daily lives as well. For example, kitchen sink’s metal water traps, sewer water pipes, fire sprinkler copper pipes, water cooling systems for large air conditioning (A/C) units, dental implants, etc. can all suffer from MIC. The well‐known Alaska pipeline leak in 2006 was due to a small 6.4 mm by 12.7 mm hole likely caused by microbes (Jacobson, 2007). This incident was the watershed moment in the growing awareness of MIC.

Many microbes in nature live in synergistic mixed‐culture biofilms. Biofilms are behind MIC because electroactive sessile cells in biofilms, not planktonic cells, can harvest electrons from energetic metals, and biofilms can harbour high concentrations of corrosive agents such as organic acids underneath. In a biofilm, the volumetric sessile cell count is easily 10^2^ or 10^3^ times higher than that of planktonic cells. Although the sessile cell number may be just a tiny fraction of the total planktonic cell number, sessile cells are usually packed in a thin, often 50–200 µm, biofilm. This alone means that a biofilm provides a different biochemical environment for corrosion.

The field environment is not sterile, allowing a diverse variety of microbes to flourish. Even in a living human being, there are up to several pounds of microbes. Some of them play important roles in our digestive system, immune system and even our mental health according to microbiome studies (Hayes *et al*., 2020). We all know that dental plaques, which are microbial biofilms, are unavoidable despite our daily brushing, flossing, and rinsing with an antiseptic mouthwash. The plaques can harbour acid producing bacteria (APB) that cause local acidity, leading to dental caries and biocorrosion of some dental alloys.

It may be futile to classify microbes into corrosive and non‐corrosive microbes. It is true that some microbes such as APB and the notorious sulphate‐reducing bacteria (SRB) are often behind MIC, many kinds of microbes can one way or another cause or accelerate corrosion. Many microbes are capable of producing organic acids and the notable ones are classified as APB, but the APB presence in a corrosion case does not imply acid corrosion at all, unless there is at least an indication that the bacteria are actually acidifying the local environment, rather than living a non‐APB lifestyle. SRB, though classified as obligatory anaerobes, are ubiquitous even in aerobic environments. When there is a significant amount of dissolved oxygen (DO), the dissimilatory reduction of sulphate, an electron acceptor for SRB, is inhibited. Some species of SRB have been observed to use O_2_ as a temporary electron acceptor in aerobic respiration without actual growth (Thauer *et al*., 2007). Once the DO in the surroundings becomes sufficiently low, sulphate respiration commences using an electron donor such as organic carbon or H_2_ for energy production.

## Examples of biocorrosion against various materials in different settings

SRB are the most studied and monitored class of organisms in MIC because they are behind many carbon steel pipeline failures in the oil and gas industry. In recent years, the science behind SRB MIC has seen tremendous advances. It is now known that H_2_S is not the culprit of SRB MIC of carbon steel when broth pH is near neutral, but rather extracellular electron transfer (EET) from elemental Fe(0) for sulphate reduction in energy production (Jia *et al*., 2019a). The two electrochemical half‐reactions in EET‐MIC of carbon steel by SRB below combined together is thermodynamically favourable (Wang *et al*., 2020),

(1) $$4\mathrm{Fe}\to {\mathrm{Fe}}^{2+}+8e^{‐}(E^{\circ }=‐447\mathrm{mV})$$

(2) $${\text{SO}}_{4}^{2‐}+9H^{+}+8e^{‐}\to {\text{HS}}^{‐}+4H_{2}O\;(E^{\circ \prime }=‐217\text{mV})$$

Thus, it is not surprising that pre‐grown mature SRB biofilms corrode more aggressively when exposed to a fresh culture medium with no or a reduced level of carbon source (Dou *et al*., 2019). A hot water tank provides a breeding ground for bacteria. Even at a temperature such as 80°C, thermophilic SRB thrive. In fact, some SRB are known to survive at 120°C (Rosnes *et al*., 1991). EET‐MIC by SRB accompanied by rotten egg smell (H_2_S) has been reported for carbon steel hot water tanks (Jia *et al*., 2018).

Cu and its alloys release trace amounts of Cu^+^ and Cu^2+^, which kill or inhibit many microorganisms, but SRB can overcome the initial inhibition by using HS^−^ to precipitate Cu ions. Very reactive SRB metabolite S^2−^, or HS^−^ (dominant sulphide species), makes the electrochemical corrosion of Cu thermodynamically favourable by forming extremely insoluble chalcocite as shown below,

(3) $$2\mathrm{Cu}+{\mathrm{HS}}^{‐}\to {\mathrm{Cu}}_{2}S+H^{+}+2e^{‐}$$

(4) $$2H^{+}+2e^{‐}\to H_{2}$$

yielding the following overall reaction for metabolite MIC (M‐MIC) of Cu (Wang *et al*., 2020),

(5) $$2\text{Cu}+{\text{HS}}^{‐}+H^{+}\to {\text{Cu}}_{2}S+H_{2}(g)(\Delta G^{\circ \prime }=‐58.3\;\text{kJ}\;{\text{mol}}^{‐1})$$

This M‐MIC can happen with or without SRB utilization of H_2_ as an electron donor using 2H^+^/H_2_ shuttle for EET. Various cases of copper piping MIC have been reported in the literature. They range from industrial heat exchanger pipes to fire sprinkler pipes, and to drinking water pipes. Drywall manufactured with dirty water has also been associated with Cu corrosion of water pipes and AC coils in homes (Hooper *et al*., 2010). In a humid environment, contaminated drywall provides a suitable environment for SRB growth because sulphate (in gypsum), organic carbon and other nutrients (in contaminants) are available. Biogenic H_2_S released by SRB explains the rotten egg smell that is associated with copper pipe corrosion by ‘toxic’ drywall. Cu can also be corroded by nitrate‐reducing bacteria (NRB) via EET‐MIC with nitrate as the terminal electron acceptor because nitrate has a much higher reduction potential than sulphate (Pu *et al*., 2020).

Many engineering metals possess their corrosion resistance because they react rapidly with O_2_ to form thin but dense passive films. However, microbes are capable of compromising the films. Various stainless steels are widely used in industrial and consumer applications because of their good corrosion resistance, which is due to a chromium oxide passive film that is instantly repaired in air if damaged. However, they are susceptible to MIC. Both SRB and NRB biofilms are found to compromise their passive films (Yu *et al*., 2020). Even titanium, one of the best corrosion‐resistant metals, is not immune to MIC pitting attacks (Khan *et al*., 2019).

The broad definition of corrosion covers the deterioration of non‐metallic materials as well. Concrete is widely used in various infrastructures due to its excellent corrosion resistance and low cost. However, MIC of sewer pipe mains is a major problem, causing the mortar on the surface to fall off or pipes to crack, thereby shortening the service life. Concrete MIC is typically caused by acidification as a result of the synergistic growth of SRB and sulphide‐oxidizing bacteria (SOB). In the liquid phase, SRB growth releases H_2_S to the gas phase, which is oxidized by O_2_ with the biocatalysis of aerobic SOB adhered to the headspace concrete surface to produce sulphuric acid (Grengg *et al*., 2017). Organic acids produced by biofilms can damage alkaline concrete.

## Perspectives

The MIC science behind pure‐culture SRB, NRB and APB is relatively mature. However, there are only limited insights into mixed‐culture MIC such as one microbe’s metabolic product serving as the carbon energy source for the other microbes, and an aerobe (or facultative microbe) providing an anoxic environment for SRB and NRB respirations. If the bulk‐fluid pH is not that acidic, acid corrosion via M‐MIC may still happen, because the true pH underneath an APB biofilm can be several units lower (Jia *et al*., 2019b). A common fallacy is to view the most abundant microbes in the surroundings as the culprit in MIC forensic analysis, and thus targeting them for treatment. Without mechanistic evidence, the abundance can only mean that the corrosion environment favours the growth of the specific species. ‘Bottom feeders’ in a biofilm that are electroactive microbes can cause EET‐MIC with just a few layers of sessile cells while other sessile cells above them, much more in cell numbers, may share the energy without directly causing MIC. Advances in microbiome will help MIC researchers better understand microbial synergy in mixed‐culture MIC.

It is necessary to investigate various MIC phenomena in different settings and dissect their underlying mechanisms. With better understanding, MIC can be minimized by proper mitigation. Biocide dosing, often combined with surface scrubbing (or pipeline pigging), is a common practice. New strategies can minimize environmental impact of biocides. Some naturally occurring D‐amino acids and nature‐inspired peptides are found to disperse biofilms. They have been investigated to enhance biocide mitigation of MIC with reduced biocide dosages (Zhu *et al*., 2019; Jia *et al*., 2019b, 2019c,2019b, 2019c). Materials themselves can also be manipulated to fight against biofilms to mitigate MIC. For example, Cu‐bearing stainless steel and Al_0.4_CoCrCuFeNi high‐entropy alloy (HEA) were invented to release metal ions that effectively inhibit some biofilms in lab studies (Zhou *et al*., 2020). These materials are permanently antimicrobial, unlike antimicrobial coatings which can wear off.

## Conflict of interest

None declared.
